# Supplementary material for: Antibiotic prescribing and antimicrobial resistance: An evaluation of clinical teachers’ knowledge, attitude and practices at a South African dental school
Source: PLoS One. 2026 Mar 24;21(3):e0344962. doi: 10.1371/journal.pone.0344962 (PMC13012509; doi:10.1371/journal.pone.0344962)
Supplement: S1 Appendix — (DOCX) [file pone.0344962.s001.docx]

**S1 Appendix : CLINICAL TEACHERS QUESTIONNAIRE**

1. Demographic questions

□ Male □ Female □ Prefer not to say

2. How many years have you been working as a dentist?

□ ………………………. years

3. Select which best describes your current employment:

□Private practitioner

□Public health

□Academics

□Part time private practice/academics

Part 1: Antibiotic Knowledge

1. Which antibiotic do you prescribe most often for an adult patient with no medical allergies?

□ Amoxicillin

□ Amoxicillin and Clavulanic acid

□ Amoxicillin and Metronidazole

□ Other, please specify …………………………….

2. If a patient is allergic to Penicillin, which antibiotic do you usually prescribe?

□ Erythromycin

□ Clindamycin

□ Azithromycin

□ Other, please specify ………………………………

3. What is the duration that you prescribe a course of antibiotics for?

□1-2 days

□3 days

□5days

□7 days

□>7days

4. What do you understand by the term Antibiotic Resistance? Please select one:

□It is an uncontrolled infection, even after taking high doses of antibiotics

□It is an inadequate dose of antibiotics that causes resistance

□It is bacteria that resist the effect of antibiotics

□It is the body that does not respond to antibiotics

5. Do you think the frequent use of antibiotics might reduce the pharmacological treatment efficacy when using the same antibiotic again in the same patient?

□Yes □No □Unsure

6. Do you think that antibiotic resistance is due to antibiotic prescription?

□Yes □No □Unsure

7. Do you believe that antibiotic resistance is of growing concern?

□Yes □No

8. What is your primary source of updated information?

□Scientifically published literature

□Continuing dental education and conferences

□Textbooks

□Internet

□Social media

9. Do you know about the AWaRe classification by WHO (World Health Organization) to use antibiotics?

□Yes □No □Unsure

10. Do you know about the WHO Global Action Plan on AMR?

□Yes □No □Unsure

Part 2 Antibiotic Prescribing Attitude

1.Are you aware of the guidelines for antibiotic prophylaxis?

□Yes □No □Unsure

2.Do you follow guidelines for antibiotic prophylaxis?

□Yes □No □Unsure

3. Which guidelines are you currently following?

□American Dental Association

□American Heart Association

□NICE (National Institute for Health and Care Excellence UK) Guidelines

□American Association of Endodontists Guidelines

□ Standard Treatment Guidelines and Essential Medicines List

□ Not sure

□Other, please specify…………………………………

4. Do you enquire from your patient whether he/she has taken a course of antibiotics in the past week before prescribing antibiotics?

□Yes □No

5. Do you advise your patients to adhere to the dosage regimen and inform them of the consequences of not doing so?

□Yes □No

6. When prescribing antibiotics, do you base your decision on: (You may select more than one choice)

□Patient symptoms

□Clinical protocols

□Cost of the antibiotic

□Clinical experience

□Other, please elaborate……………………

7. What are the non-clinical factors that influence your antibiotic prescription? (You may select more than one choice)

□Patient’s preference

□Knowledge obtained during undergraduate course

□Availability at pharmacy

□Recommendation from expert colleagues

□International guidelines

□ Unavailable appointment for several weeks

□Other, please elaborate…………………….

8. Do you believe that your undergraduate education adequately prepared you to effectively prescribe antibiotics and practice antimicrobial stewardship?

□Yes □No □Unsure

Part 3 Antibiotic Prescribing Practice

1. Is it common to prescribe antibiotics in your daily dental practice?

□Yes □No □Unsure

2. For which of the following clinical situations do you prescribe antibiotics for: (You may select more than one choice)

□Pain Relief

□Difficulty in obtaining anaesthesia

□Reversible pulpitis

□Irreversible pulpitis

□Root canal treatment

□ Facial swelling

□Abscess of dental origin with fever

□Localized dentoalveolar abscess

□Localized dentoalveolar abscess with draining fistula

□Simple extractions

□Surgical extractions

□Immediate dentures

□Non-surgical periodontal treatment

□Acute gingivitis and stomatitis

□Implant placement

□Increased risk of infective endocarditis

3. Which medical condition/s do you consider prescribing antibiotic prophylaxis to, in order to avoid infective endocarditis? (You may select more than one choice)

□Diabetes mellitus

□Autoimmune diseases

□Immunosuppressive therapy

□HIV/AIDS

□Rheumatic heart disease

□Heart bypass surgery

□Pacemaker

□Infective endocarditis

□Heart defects

□Joint prostheses

4. If antibiotic prophylaxis is indicated, what dosage do you prescribe?

□Amoxicillin 2G - 1 hour before treatment

□Amoxicillin 1G - 1 hour before treatment

□Amoxicillin 1G - 1 hour before and 6 hours after

5. How many patients have you prescribed antibiotics to in the last two weeks?

□None □1-4 □5-7 □ >7
