# Supplementary material for: Antibiotic prescribing and antimicrobial resistance: An evaluation of clinical teachers’ knowledge, attitude and practices at a South African dental school
Source: PLoS One. 2026 Mar 24;21(3):e0344962. doi: 10.1371/journal.pone.0344962 (PMC13012509; doi:10.1371/journal.pone.0344962)
Supplement: Attachment — Submitted filename: Plos One Letter for Rebuttal 20 Jan 2026.pdf [file pone.0344962.s003.pdf]

UNIVERSITY of the WESTERN CAPE

FACULTY of DENTISTRY
UNIVERSITY of the WESTERN CAPE

The Editor
PLOS ONE

20 January 2026

Dear Editor

Re: Resubmission of manuscript PONE-D-25-62403
Antibiotic Prescribing and Antimicrobial Resistance: An Evaluation of Clinical Teachers' Knowledge, Attitude and Practices at a South African Dental School

Thank you for your feedback and recommendations regarding our submitted manuscript. The added insights have been helpful in managing the requested revisions. I have tabulated all feedback and the revisions implemented in response to the peer reviewers' comments (please see below). All changes to the manuscript have been highlighted in blue. Specific questions and information requested by reviewers have been addressed within the tabulated form as well.

Funding for part of this study was supported by the South African Medical Research Council Self-Initiated Research Grant. This is the title of the grant; no grant number is allocated.

No authors received salaries from this grant.

I trust that we have adequately addressed all required edits and comments. I hope that you will view our revised manuscript favorably.

Kind regards

Dr Suwayda Ahmed
Senior Lecturer
Department of Prosthodontics
Faculty of Dentistry
Tel: (021) 9373091
suahmed@uwc.ac.za

FEEDBACK	ACTION
Reviewer 1	
1. Methodology Requires Further Clarification.<br>The sampling method is clearly described, but justification for using convenience sampling should be added, including its limitations.<br>Provide more details on questionnaire validation, including:<br>Specific changes made after the pilot study<br>Reliability statistics, if available<br>Please clarify if the questionnaire was adapted from validated tools or fully developed by the authors.	Acknowledged.<br>The methods section has been edited and all recommended feedback has been addressed.<br>Lines 169-172<br>Lines 186-194<br>Lines 196-197<br>Lines 201-203<br>Lines 206-209
2. Statistical Analysis Section Needs Strengthening<br>Although descriptive statistics and chi-square tests are mentioned, no statistical test results (p-values, test statistics, effect sizes) are presented in the Results.<br>The authors must:<br>Add p-values for associations examined.<br>Indicate which variables showed statistically significant relationships.<br>Provide a brief interpretation of each.	Acknowledged.<br>This has been revised in the results section. Please see highlighted text in results section.
3. Results Section Should Be More Analytical<br>Currently, results are descriptive. To enhance scientific value:<br>Include comparative analysis (e.g., years of experience vs.	Acknowledged.<br>The results section has been reworked to address the recommended feedback. All changes are highlighted in blue.<br>Lines 237-238<br>Lines 241-338

prescribing behaviour).<br>Present findings from chi-square analyses.<br>If no statistically significant relationships were found, state this explicitly.	
4. Discussion Requires Deeper Critical Interpretation<br>The discussion is informative but:<br>It should integrate statistical associations (once added).<br>The issue of clindamycin overuse must be expanded with discussion of:<br>Adverse outcomes<br>International stewardship recommendations<br>Consider adding regional comparisons (Africa) in addition to global literature.	Acknowledged.<br><br>The discussion has been substantially reworked.<br>Regional comparisons have been included.<br>Lines 340-458
5. Limitations Section Needs Expansion<br>Include:<br>Sampling bias from convenience sampling<br>Self-report bias<br>Single-institution limitations<br>Potential non-responder bias	Acknowledged.<br>The Limitations section has been rewritten to take all the recommended points into consideration.<br>Lines 478-484<br>Lines 489-492
6. Language and Consistency<br><br>The manuscript is well written but requires:<br>Minor grammatical refinement<br>Consistency in terminology (e.g., “antibiotic prescribing patterns” vs “prescribing practices”)<br>Ensure all acronyms are spelled out at first mention.<br>Add citations for WHO AWaRe, national guideline documents, and recent AMS literature (optional but recommended).	Acknowledged.<br>Prescribing practices used throughout the manuscript<br>Acronyms spelled out<br>Manuscript has been edited for grammatical errors.<br>Citations provided for WHO AWaRe, South African national guideline documents (References 16, 23 and 24)<br><br>Lines 229 and 296

Figure 1 should include axis labels and clearer formatting. Ensure tables follow PLOS ONE formatting guidelines.	
Reviewer 2	
Although the manuscript includes a Limitations section, these aspects should be more acknowledged in the Discussion, particularly: the single-institution sample limits generalizability, convenience sampling, and self-reported prescribing practices.	Acknowledged.<br>The limitation section has been rewritten.<br>Lines 478-492
Please provide more discussion on the high dependence on clindamycin as the penicillin alternative. And whether this aligns with the emerging guidelines for antibiotics?	Acknowledged.<br>More detail provided in the discussion and has been rewritten.<br>Lines 340-458
Certain results are reported in both tables and text, leading to unnecessary repetition.	Acknowledged.<br>The results section has been edited and reworked.<br>Lines 241-338
4-Minor typographical inconsistencies are present.	Acknowledged.<br>Manuscript has been edited for grammatical errors.
Reviewer 3	
Abstract - misses some really important information - as per the point above - and in the methods section (e.g. when was this survey undertaken and in which dental school) - and doesn't make efficient use of word count e.g. by repeating points between the results (last line) and conclusion (first line)	Acknowledged.<br>The abstract has been rewritten to address reviewers' feedback.<br>Lines 67-72<br>Lines 78-79<br>Lines 98-100
Introduction - is too long so looses the power of its key points	Acknowledged<br>The introduction has been reorganised and unnecessary detail has been removed.
Methods & results - ensure you cover all the points from the CROSS guideline.	The methods section has been edited to address feedback and follow CROSS guidelines<br>The result section has been edited and certain areas have been rewritten.<br>Lines 291-338
Discussion - as per comments on the introduction. Much of the first three paragraphs would fit better in the abstract and/or	Acknowledged<br>Paragraphs have been moved to the introduction.<br>More detail and explanation have been provided for cited studies.

introduction. In the third paragraph it's unclear why so much emphasis is placed on awareness of the WHO Global Action Plan. Clinical teachers certainly need to be aware of how it is has been translated into their context, but the need for awareness of the policy document seems curious. I have not gone through the discussion in detail because it really needs a significant redraft. When doing that, please remember to provide the reader with more information about key papers - for example in which countries were the studies by Sutej and by Hirayama undertaken? And in the next paragraph - it leaves the reader hanging. You need to provide more detail about how the cited studies vary rather than just citing 3 studies.	Explanation for WHO Global Action Plan, South African National AMR Framework has been provided in discussion.<br>The discussion has been reworked.<br>Lines 340-458
